# The Lack of Evidence for an Association between Cancer Biomarker Conversion Patterns and CTC-Status in Patients with Metastatic Breast Cancer

**DOI:** 10.3390/ijms21062161

**Published:** 2020-03-21

**Authors:** Stefan Stefanovic, Thomas M. Deutsch, Sabine Riethdorf, Chiara Fischer, Andreas Hartkopf, Peter Sinn, Manuel Feisst, Klaus Pantel, Michael Golatta, Sara Y. Brucker, Marc Sütterlin, Andreas Schneeweiss, Markus Wallwiener

**Affiliations:** 1 Department of Gynecology and Obstetrics, Mannheim University Hospital, University of Heidelberg, Theodor-Kutzer-Ufer 1-3, 68167 Mannheim, Germany; 2 Department of Gynecology and Obstetrics, Heidelberg University Hospital, Im Neuenheimer Feld 440, 69120 Heidelberg, Germanymarkus.wallwiener@med.uni-heidelberg.de (M.W.); 3 Institute of Tumor Biology, University Hospital Hamburg-Eppendorf, Martinistraße 52, 20246 Hamburg, Germany; 4 Department of Women’s Health, University Hospital Tübingen, Calwerstraße 7, 72076 Tübingen, Germany; 5 Institute of Pathology, Heidelberg University Hospital, Im Neuenheimer Feld 224, 69120 Heidelberg, Germany; 6 Institute of Medical Biometry and Informatics, University of Heidelberg, Im Neuenheimer Feld 130.3, 69120 Heidelberg, Germany; 7 Department of Obstetrics and Gynecology, University of Tübingen, Calwerstraße 7, 72076 Tübingen, Germany; 8 National Center for Tumor Diseases (NCT) Heidelberg, Im Neuenheimer Feld 460, 69120 Heidelberg, Germany; 9 German Cancer Research Center (DKFZ), Heidelberg, Im Neuenheimer Feld 280, 69120 Heidelberg, Germany

**Keywords:** breast cancer, intrinsic subtype, biomarker conversion, circulating tumor cells, liquid biopsy

## Abstract

Circulating tumor cell (CTC) detection is a prognostic factor in the metastatic breast cancer (MBC) setting. Discrepancies in primary (PT) and metastatic tumor (MT) genetic profiles are also of prognostic importance. Our study aimed to compare the CTC statuses and prognoses between those with subtype stable MBCs and MBCs with specific biomarker conversions. The study enrolled 261 MBC patients, treated at the National Center for Tumor Diseases, Heidelberg, Germany in a five-year period. All underwent PT and MT biopsies and subsequent CTC enumeration before the initiation of systemic therapy. ER and HER2 statuses of the PTs and MTs were determined and progression free survivals (PFSs) and overall survivals (OSs) were recorded. We compared CTC statuses, CTC counts, PFSs and OSs between subgroups of patients with different receptor change patterns. Patients who had tumors that converted to triple negative MTs had the shortest median OSs, while HER2 expression was not associated with a shorter median OS. No significant differences in PFSs and OSs have been demonstrated by Kaplan-Meier curve comparisons in any of the subgroup analyses. CTC counts were similar in all subgroups. CTCs were comparably less frequently detected in patients with a stable HER2 expression. Similar proportions of CTC positives were observed in all other subtype change pattern subgroups, barring the aforementioned HER2 stable subgroup. The detection of CTCs was of no appreciable prognostic value in different receptor change pattern subgroups in our cohort.

## 1. Introduction

Breast cancer (BC) is the most common female tumor both worldwide and in Germany [1,2,3]. It is also the most common cause of cancer-related death in women [1]. An estimated 8% of patients with breast cancer in Germany are diagnosed with metastatic disease (MBC) [4]. Also, up to 11% of German patients with BC suffer a metastatic recurrence within 10 years of initial treatment [5].

Systemic treatment is a therapeutic mainstay in patients with MBC. Overall survival and progression-free survival have been prolonged upon the introduction of molecularly targeted agents like HER2 specific antibodies, endocrine therapies, immunotherapies and PIK3CA, PARP, CDK4/6 and mTOR inhibitors. Despite these considerable innovations, the prognosis of MBC remains poor [6]. Nevertheless, improvements in survival have been made, and ongoing efforts are aimed at defining additional prognostic and predictive factors, as well as advancing the concept of individualized therapy in the context of MBC [7,8]. Some of the most promising avenues of research have led to the recognition of the prognostic and predictive values of circulating tumor cells (CTCs) and receptor discrepancy (a change in receptor expression) between the primary (PT) and metastatic tumor (MT) [9,10,11,12,13,14,15,16,17].

CTC detection in the peripheral blood has been demonstrated as a prognostic factor by multiple authors in the MBC setting [18,19,20]. Whether they are of predictive value remains open to discussion and requires further study [18]. In any case, since metastatic breast cancer is a heterogeneous entity, some researchers suggest that the detection of CTCs can provide a non-invasive modality of optimizing treatment decisions, justifying the name “liquid biopsy” [11,21,22].

It has long been recognized that tumor receptor expression (i.e., genetic profile) dictates its clinical behavior, treatment success, and prognosis [23,24,25,26]. Subsequently, discrepancies in the genetic profiles of PT and MT were observed leading to the recommendation that therapy should be tailored with the metastatic tumor’s biology in mind [10,13,27]. Logically, it was demonstrated that receptor conversion (i.e., tumor biomarker change) between the PT and MT is of paramount importance for prognostication and therapy selection [9,14,15,16,17,28].

The current study aimed to compare the CTC counts and CTC status between tumor subtype converters and stable MBC patients as well as between the particular conversion patterns. Since, as elaborated previously, the results of multiple studies suggest that certain receptor conversions between the PTs and MTs are associated with adverse outcomes, we posit that patients harboring tumors with converted tumor subtypes should have an elevated CTC count, since it is also an independent negative prognostic factor.

## 2. Results

The study enrolled 261 patients with MBC, with CTC detected in blood specimens from 90 (34.5%) of them. Sixty-two patients (23.8%) had a single metastatic site, while 199 (76.2%) had metastases involving multiple sites. Bones were involved in 153 (58.6%) and viscera in 213 patients (81.6%).

Primary tumors were ER positive in 192 (73.6%) and HER2 positive in 51 patients (19.5%) while the same was true for metastatic tumor biopsies in 182 (69.7%), and 52 patients (19.9%), respectively. Receptor conversion between PT and MT was recorded in 61 patients (23.4%).

Differences between patients with stable receptor expression across PT and MT and those with receptor conversion are represented in Table 1. A significantly lower proportion of PTs and MTs was HER2 positive in the subgroup with stable molecular subtype both for PT 27 (13.5%) versus 24 (39.3%) (*p* < 0.001, Table 1) and for MT 27 (13.5%) versus 25 (41%) (*p* < 0.001, Table 1). Conversely, receptor stable MTs were significantly more frequently ER positive than the converters: 151 (75.5%) versus 31 (50.8%); *p* < 0.001 (Table 1). No differences in CTC status, CTC counts, PFS or OS were observed between the two subgroups.

**Figure 1:**
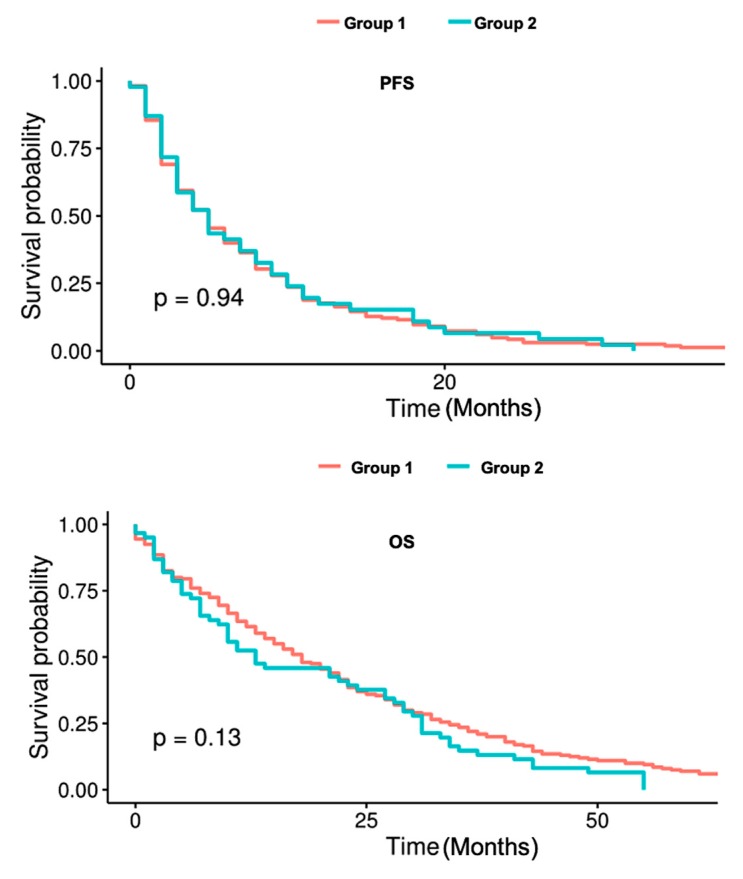
PFS and OS comparison between patients with stable (Group 1) and converted tumor subtype (Group 2).

Time to event analyses using Kaplan-Meier curves and Log-Rank test revealed no significant differences between the receptor stable and the receptor converter group regarding PFS (*p* = 0.94) and OS (*p* = 0.13) as represented in Figure 1.

**Figure 2:**
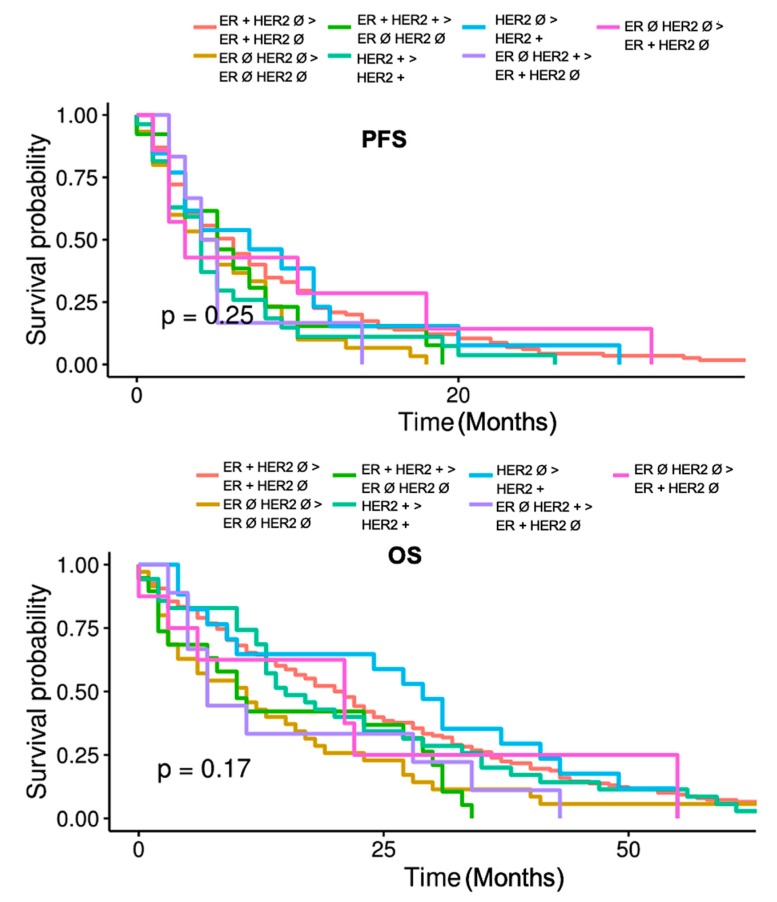
PFS and OS comparison between seven subgroups based on specific receptor change or stable patterns between PTs and MTs. Ø—negative; ER—estrogen receptor; HER2—human epidermal growth factor receptor 2.

Specific receptor conversion patterns between PTs and MTs allow for further subdivision of the study population into 7 distinct subgroups. The subgroups were formed according to the clinical relevance of the particular biomarker conversion patterns for therapeutic decision making, foremost loss, or upregulation of target structures for endocrine or antibody treatment. Table 2 delineates differences between the subgroups. The criteria for subgroup division appear to be appropriate since the subgroups differ significantly in their CTC-positive share (*p* = 0.04, Table 2). The final three subgroups represented in Table 2 have higher than expected proportions of patients who are CTC positive. On the other hand, patients with a stable HER2 positive phenotype were shown to have had a lower proportion of CTC positives. Even though it was not found to be statistically significant, patients with conversion from ER negative HER2 positive to ER positive HER2 negative and those with conversion from an ER positive HER2 positive PT to a triple negative MT and triple negative subtype stable patients had the shortest OSs (Groups 2, 3, and 6 when counted top to bottom in Table 2). Figure 2 demonstrates Kaplan-Meier curves for PFS and OS in the seven subgroups. No significant differences between the subgroups in this context were demonstrated.

**Figure 3:**
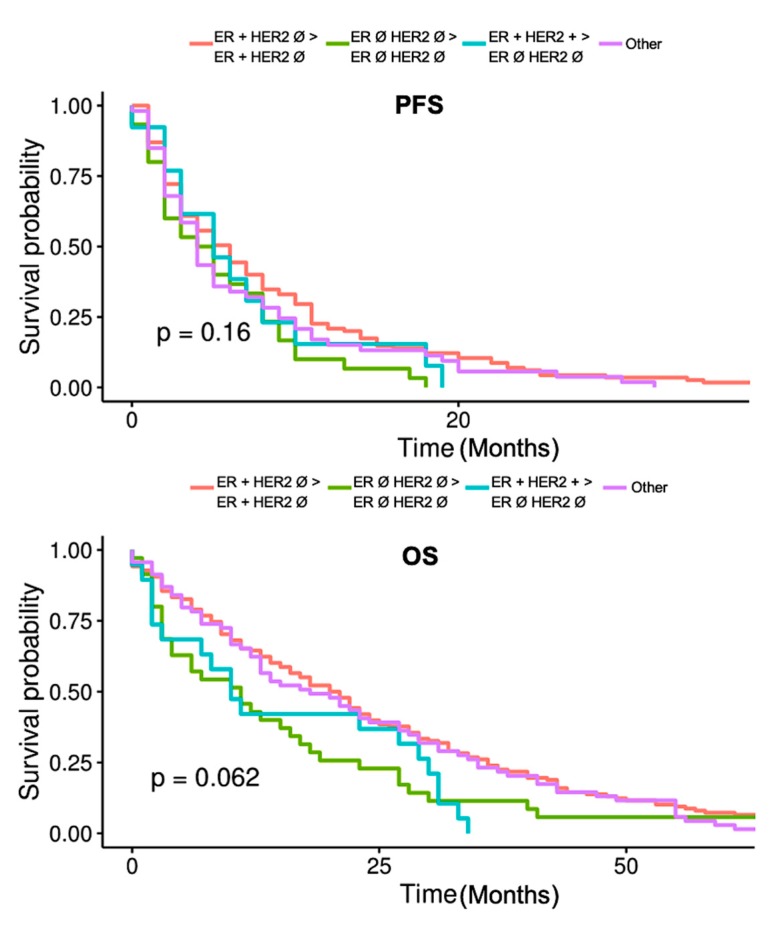
PFS and OS comparison between four patient subgroups based on therapeutically relevant receptor change or stable patterns between PTs and MTs. Ø—negative; ER—estrogen receptor; HER2—human epidermal growth factor receptor 2.

Comparisons of the characteristics of four patient subgroups demonstrating therapeutically relevant receptor evolution between PTs and MTs are represented in Table 3. Bone metastases were less common in the triple negative receptor stable subgroup. The forth subgroup designated “Other” had a lower proportion of CTC positive patients (21.7%) compared to the other three subgroups which all had approximately 40% of CTC positive patients. However, the differences between the groups were found to be statistically non-significant (*p* = 0.08). The two subgroups in which the MT was triple negative (especially the one in which the PT was ER and HER2 positive) had a shorter OS compared to the other two subgroups. Interestingly, PFS was similar between the subgroups. No significant differences in PFS and OS have been demonstrated by Kaplan-Meier curve comparisons using the Log-Rank test (see Figure 3).

## 3. Discussion

In this study, we analyzed the CTC counts and CTC status, comparing tumor subtype converters, stable MBC patients and the particular conversion patterns.

Our study demonstrated no significant difference in the number of CTCs or CTC status in the peripheral blood between patients with stable and converted tumor subtypes when these subgroups were analyzed disregarding the particular patterns of receptor change. A significant difference that we observed was that patients who had undergone receptor conversion were more likely to have had a HER2 positive PT. Also, receptor conversion was more likely to lead to the development of a MT that was ER negative. Similar findings were published by other authors [9,16,29]. We could not demonstrate a statistically significant difference in PFS and OS, between patients in the stable and converted tumor subtype subgroups.

Nonetheless, when we analyzed specific receptor conversions, differences between the seven subgroups in OSs became evident, even though statistical significance was not achieved. Given our relatively small cohort and study power, the true differences in OSs between these subgroups are conceivably significant. Progress free survival was not significantly different between the subgroups. CTC positivity was significantly different between the seven subgroups (*p* = 0.04).

Even though detecting a HER2 positive tumor has historically been considered prognostically disadvantageous, our study was able to show no association between HER2 positive tumors with stable receptor expression and a poor prognosis [30]. Furthermore, tumors consistently expressing HER2, through their evolution in an individual patient, were associated with a low probability of CTC positivity in our study. Both these observations could conceivably be a consequence of the availability and widespread use of HER2 antagonists and their effectiveness against CTCs [7,8,12,31,32]. Indeed, Wallwiener et al. have demonstrated that about 74% of patients who have tumors (PTs or MTs) expressing HER2, express HER2 on CTCs, as well possibly making them susceptible to HER2 antagonists [31].

It has long been recognized that MT HER2 expression in a patient with a HER2 negative PT is also a poor prognostic sign, yet it was not associated with a poor outcome in our study, despite the fact that a high percentage of such patients in our study was CTC positive – the cause is possibly the same as stated in the previous paragraph [25]. We must, however, emphasize that, in our study, the proportion of CTC positive patients was not higher than that observed in other subgroups (HER2 stable patients excluded).

Triple negative to luminal conversion was also associated with a high percentage of CTC positives in our study but the overall survival was comparably good. The reason is, perhaps, the efficacy of modern systemic therapy [7,8]. The proportion of CTC positive patients was also not different from other subgroups save the HER2 stable patients.

Interestingly, patients who have had cancer, who converted from ER negative HER2 positive PT to a luminal HER2 negative subtype MT, had a poor prognosis. These findings are concordant with other data which have demonstrated that conversion from an ER negative PT to an ER positive MT has been associated with reduced OS [14]. In this study, CTC positives were observed with a similar frequency, compared to other subgroups, not considering patients with HER2 stable tumors.

There seems to be a statistically significantly higher frequency of CTC positive patients in the luminal subtype receptor stable and triple negative receptor stable patients and luminal to triple negative converters compared to all other patients in our cohort (Table 3). OSs reflect the fact that CTCs are associated with poor overall survival with the notable exception of longer OSs in the luminal subtype receptor stable subgroup. Again, this is probably due to the efficacy of modern systemic therapy.

It would seem that receptor conversion was not a poor prognostic factor per se. Rather, the type of receptor conversion taking place provided more prognostic utility [9,14,16]. In addition, some aggressive tumor subtypes like the triple negative subtype were associated with a decreased OS even if receptor expression remained stable, in both our and other studies [10,24]. CTCs were not found more frequently in these patients either.

It would seem that CTC counts and proportions of CTC positive patients do not differ in a clinically meaningful way between the subgroups analyzed. in our study, which is somewhat surprising given that differences in overall survival were observed. This could be a consequence of the relatively low number of patients presenting with some subtype conversion patterns in our study. Thus, there is a possibility that a true effect has been missed and might become evident once our patient cohort reaches a larger size in the future. Also, no significant differences in OSs were observed between subtype converters as a whole (disregarding specific conversion patters) and subtype stable patients. Whether our study cohort is representative of the true population of MBC patients remains to be elucidated. Also, the tumor subtypes were determined utilizing the IHC and FISH methodologies, which might yield different results when compared with PCR based techniques [33].

Bone metastases were significantly less likely to occur in ER negative patients, an observation made by multiple authors thus far [29,34]. Yet, MT in the bone was not associated with HER2 expression in the current study in stark contrast to the results we have had published previously [29].

## 4. Materials and Methods

### 4.1. Study Design

This was a retrospective, single-center, cohort study. The study was conducted at the National Center for Tumor Diseases (NCT), Heidelberg, Germany in accordance with the regulations of the tissue bank and the approval of the ethics committee of the Medical Faculty Heidelberg of the Heidelberg University, approval No. S-295/2009 issued on 19 November 2009. 

### 4.2. Patients

All adult patients that had been treated for metastatic breast cancer between March 2010 and May 2015 were assessed for study enrollment eligibility. All patients with measurable metastatic disease regardless of its localization, with executed biopsy for both PT and MT and written informed consent for study participation were included in the study. In order for a patient to be enrolled, a blood sample for CTC enumeration had to have been collected within 12 months of the biopsies. Patients lost to follow-up were excluded from the study.

### 4.3. Therapy

All patients were treated according to the German national guidelines and NCT SOPs [35,36]. Therapeutic decisions were influenced by receptor expression of the MT. Treating physicians were blinded to the CTC status.

### 4.4. PFS and OS

Therapy response was evaluated every 3 months via CT and/or MRI scan and categorized via Response Evaluation Criteria in Solid Tumors (RECIST) into progressive disease (PD), stable disease (SD), complete remission (CR) or partial response (PR) [37]. All radiologists performing imaging studies were blinded to the patient’s treatment regimen, CTC status and tumor subtypes. Overall survival was measured in months since the moment of study enrollment (at the time of the MT biopsy) and it pertained to all-cause mortality. Both PFS and OS were recorded until death, study conclusion or loss to follow-up.

### 4.5. CTC Enumeration

Blood for CTC enumeration was sampled before the initiation of a new line of systemic therapy. In detail, 7.5 ml of whole blood from a peripheral vein were collected in a CellSave tube (Menarini, Silicon Biosystems, Bologna, Italy). These samples were stored at room temperature for <96 h before being analyzed using the Cell-Search^TM^ assay (CellSearch^TM^ Epithelial Cell Kit/CellSpotter^TM^ Analyzer, Menarini, Bologna, Italy). Sample processing and analysis were conducted according to the manufacturer’s instructions. CTC detection was performed by trained staff and independent reviewers confirmed the CTC enumeration results. CTC counts higher than 5 CTC per 7.5 mL of peripheral blood were considered CTC positive [38]. All investigators and technical staff involved in CTC enumeration were blinded to the patient medical history and treatment regimen.

### 4.6. Receptor Status Assessment

ER, PR, and HER2 receptor statuses were collected from medical records. Analysis were performed at the Heidelberg University Hospital and in some instances at peripheral hospitals and defined as hormone receptor positive if ER was measured with an Allred score of ≥ 3/8 or IRS ≥ 3/12. HER2 status was assessed by immunohistochemistry (IHC) and/or fluorescent in situ hybridization (FISH). HER2 status was positive when the ISH score was either 3+ or the ISH score was +2 with positive fluorescence in situ hybridization (FISH) or chromogenic in situ hybridization (CISH) staining [33]. All technicians and pathologists were blinded to the patient’s medical history, therapy, and CTC status. We did not analyze PR receptor conversions in this study. This was a conscious choice on our part, attempting to diminish the possible number of PT-MT receptor combinations. As HER2 conversion and ER conversion have been studied more extensively than PR conversion we chose to omit it from the analyses.

### 4.7. Data Collection and Analysis

Demographic data and clinical characteristics were described as frequency and percentages for count data and median and range for continuous data. Three different sets of subgroup analyses were conducted in our study. Firstly, we compared CTC status, CTC count, PFS and OS between patients with a stable receptor expression (Group 1) and those with receptor conversion (Group 2). Secondly, we compared CTC status, CTC count, PFS and OS between seven subgroups created according to the specific type of receptor conversion. In the third stage, we compared CTC status, CTC count, PF, and OS between four subgroups corresponding to therapeutically relevant structured receptor changes and receptor stability. These differences between the subgroups were examined utilizing Wilcoxon rank sum tests, Kruskal-Wallis tests and Chi-squared tests, as appropriate. In addition, Kaplan–Meier plots were used to represent PFS and OS data and a Log-Rank test was used to compare the between subgroup differences in PFS and OS. Due to the exploratory character of the study *p*-values have to be interpreted in a descriptive sense and no imputation of missing data was performed. All analyses were performed using R (version 3.6.1, R Foundation for Statistical Computing, Vienna, Austria).

## 5. Conclusions

Subtype conversion patterns analyzed in this study did not seem to be associated with clinically meaningful differences in CTC counts and the proportion of CTC positive patients. However, MBC prognoses were worse in patients with triple negative MTs, in both the receptor converters and the subtype stable patients. The same can be said of our patients who have had tumors that acquired ER and lost HER2 receptors through mutation.

## Figures and Tables

**Table 1:** Demographic and clinical characteristics, CTC status, CTC count, PFS and OS according to tumor subtype conversion.

Statistics	Stable Subtype	Subtype Converters	*p*
Total, n (%)	200 (100%)	61 (100%)	
CTC positive, n (%)	70 (35%)	20 (32.8%)	0.75
CTC count, median (range)	1 (0–200,000)	0 (0–840)	0.54
Age at initial diagnosis, median (range)	50 (23–87)	48 (28–69)	0.15
Age at enrollment, median (range)	56 (24–87)	56 (30–78)	0.31
ER positive PT, n (%)	151 (75.5%)	41 (67.2%)	0.2
HER2 positive PT, n (%)	27 (13.5%)	24 (39.3%)	<0.001
ER positive MT, n (%)	151 (75.5%)	31 (50.8%)	<0.001
HER2 positive MT, n (%)	27 (13.5%)	25 (41%)	<0.001
Number of metastatic sites			0.87
One site, n (%)	48 (24%)	14 (23%)	
Multiple sites, n (%)	152 (76%)	47 (77%)	
Site of metastasis			
Bone, n (%)	118 (59%)	35 (57.4%)	0.82
Visceral, n (%)	163 (81.5%)	50 (82%)	0.93
Metastatic therapy			
First line, n (%)	25 (12.5%)	10 (16.4%)	0.41
Second line, n (%)	66 (33%)	15 (24.6%)	
Other, n (%)	109 (54.5%)	36 (59%)	
Metastatic chemotherapy			
First line, n (%)	62 (31%)	16 (26.2%)	
Second line, n (%)	64 (32%)	14 (23%)	0.15
Other, n (%)	74 (38%)	31 (50.8%)	
PFS, median (range)	5 (0–76)	5 (0–32)	0.88
OS, median (range)	18 (0–94)	13 (0–55)	0.33

**Table 2:** Comparing CTC status, CTC count, and PFS and OS between subgroups defined by specific patterns of tumor subtype conversion.

Statistics	Total, n (%)	CTC Positive, n (%)	CTC Count, Median (Range)	PFS, Median (Range)	OS, Median (Range)
Receptors (PT > MT)					
ER + HER2 Ø > ER + HER2 Ø	138 (100%)	53 (38.4%)	1 (0–200,000)	6 (1–76)	20.5 (0–94)
ER Ø HER2 Ø > ER Ø HER2 Ø	35 (100%)	14 (40%)	1 (0–74)	4.5 (0–18)	11 (0–94)
ER + HER2 + > ER Ø HER2 Ø	19 (100%)	8 (42.1%)	3 (0–840)	5 (0–19)	10 (0–29.5)
HER2 + > HER2 +	35 (100%)	3 (8.6%)	0 (0–100)	4 (0–26)	15 (0–92)
HER2 Ø > HER2 +	17 (100%)	5(29.4%)	0 (0–91)	7 (1–30)	29 (4–55)
ER Ø HER2 + > ER + HER2 Ø	9 (100%)	4(44.4%)	0 (0–840)	4.5 (2–14)	7 (3–43)
ER Ø HER2 Ø > ER + HER2 Ø	8 (100%)	3(37.5%)	3 (0–22)	3 (1–32)	21 (0–55)
*p*		0.04	0.23	0.6	0.07

Ø—negative; ER—estrogen receptor; HER2—human epidermal growth factor receptor 2.

**Table 3:** Comparisons of demographic and clinical characteristics as well as CTC status, CTC count, and PFS and OS between 4 subgroups corresponding to therapeutically relevant receptor dynamics.

Statistics	ER + HER2 Ø > ER + HER2 Ø	ER Ø HER2 Ø > ER Ø HER2 Ø	ER + HER2 + > ER Ø HER2 Ø	Other	*p*
Total, n (%)	138 (100%)	35 (100%)	19 (100%)	69 (100%)	
CTC positive, n (%)	53 (38.4%)	14 (40%)	8 (42.1%)	15 (21.7%)	0.08
CTC count, median (range)	1 (0–200,000)	1 (0–74)	3 (0–840)	0 (0–840)	0.10
Age at initial diagnosis, median (range)	50 (43–87)	51 (33–68)	44 (33–66)	48 (23–69)	0.008
Age at enrollment, median (range)	59 (24–87)	52 (35–74)	50 (35–78)	53 (29–63)	0.005
Number of metastatic sites					0.70
One site, n (%)	31 (22.5%)				
Multiple sites, n (%)	107 (77.5%)				
Site of metastasis					
Bone, n (%)	93 (67.4%)	11 (31.4%)	11 (57.9%)	38 (57.9%)	0.002
Visceral, n (%)	113 (81.9%)	29 (82.9%)	15 (78.9%)	56 (81.2%)	0.99
Metastatic therapy					
First line, n (%)	18 (13%)	4 (11.4%)	4 (21.1%)	9 (13%)	0.4
Second, n (%)	45 (32.6%)	14 (40%)	7 (36.8%)	15 (21.7%)	
Other, n (%)	75 (54.3%)	17 (48.6%)	8 (42.1%)	45 (65.2%)	
Metastatic chemotherapy					
First line, n (%)	48 (34.8%)	8 (22.9%)	6 (31.6%)	16 (23.2%)	0.048
Second, n (%)	45 (32.6%)	12 (34.3%)	7 (36.8%)	14 (20.3%)	
Other, n (%)	45 (32.6%)	15 (42.9%)	6 (31.6%)	39 (56.6%)	
PFS, median (range)	6 (1–76)	4.5 (0–18)	5 (0–19)	4 (0–32)	0.6
OS, median (range)	20.5 (0–94)	11 (0–94)	10 (0–29.5)	18 (0–92)	0.07

Ø—negative; ER—estrogen receptor; HER2—human epidermal growth factor receptor 2.
